# Treatment persistence in paediatric-onset multiple sclerosis: A Swedish nationwide registry study

**DOI:** 10.1177/13524585261448789

**Published:** 2026-05-31

**Authors:** Fredrik Sandesjö, Kyla A McKay, Katharina Fink, Fredrik Piehl, Ronny Wickström

**Affiliations:** Neuropediatric Unit, Astrid Lindgren Children’s Hospital, Karolinska University Hospital, Stockholm, Sweden; Department of Women’s and Children’s Health, Karolinska Institutet, Stockholm, Sweden; Department of Clinical Neuroscience, Karolinska Institutet, Stockholm, Sweden; Center for Molecular Medicine, Karolinska Institutet, Stockholm, Sweden; Department of Clinical Neuroscience, Karolinska Institutet, Stockholm, Sweden; Department of Clinical Neuroscience, Karolinska Institutet, Stockholm, Sweden; Center for Molecular Medicine, Karolinska Institutet, Stockholm, Sweden; Neuropediatric Unit, Astrid Lindgren Children’s Hospital, Karolinska University Hospital, Stockholm, Sweden; Department of Women’s and Children’s Health, Karolinska Institutet, Stockholm, Sweden

**Keywords:** Disease-modifying therapies, real-world evidence, children, cohort study

## Abstract

**Background:**

Comparative studies in paediatric-onset multiple sclerosis (PoMS) that also include highly effective monoclonal antibody therapies are rare.

**Objective:**

To compare treatment persistence, as a proxy of effectiveness and tolerability, across disease-modifying therapies (DMTs).

**Methods:**

Nationwide cohort study using Swedish MS Registry data on individuals with MS onset before age 18 from 2000 to 2024. Treatment persistence was analysed using Kaplan–Meier and Cox models, adjusted for demographics and treatment epoch.

**Results:**

We included 383 individuals (69.2% female; median onset age 15.8 and 17.2 years at first DMT start), observed for a median 10.1 years (interquartile range (IQR): 5.5–15.5), yielding 934 treatment episodes: rituximab (272), injectables (263), natalizumab (248), fingolimod (86) and dimethyl fumarate (65). Median age at any treatment initiation, regardless of treatment sequence, was 18.9 years, with a median Expanded Disability Status Scale (EDSS) score of 1.5 (IQR: 0.0–2.0). Treatment persistence never dropped below 50% for rituximab, while median persistence was 35.6 months for natalizumab (95% confidence interval (CI): 28.8–44.9), 34.7 for dimethyl fumarate (95% CI: 21.7–56.9), 32.0 for fingolimod (95% CI: 21.1–48.1) and 17.6 for injectables (95% CI: 14.4–20.8). Compared to injectables, adjusted hazard ratios (HRs) for discontinuation were significantly lower for rituximab (0.09; 95% CI: 0.07–0.12), natalizumab (0.39; 95% CI: 0.31–0.48), fingolimod (0.41; 95% CI: 0.30–0.55) and dimethyl fumarate (0.49; 95% CI: 0.36–0.68).

**Conclusion:**

Rituximab displayed the highest treatment persistence, supporting B-cell depletion as a first-line option in PoMS.

## Introduction

At the group level, paediatric-onset multiple sclerosis (PoMS) exhibits more inflammatory disease activity than adult-onset multiple sclerosis (MS) manifesting as higher relapse rates and lesion loads detected on magnetic resonance imaging (MRI).^1^ It can thus be argued that the early initiation of highly effective disease-modifying therapies (DMTs) is especially important in this age group.^2–4^ Nevertheless, traditional moderate-efficacy DMTs (interferons and glatiramer acetate) remain the preferred option among the majority of clinicians treating PoMS.^5,6^ This conservative approach likely reflects the paucity of evidence from randomised controlled trials, with few DMTs specifically approved for PoMS.^1^ Only relatively recently have the first three paediatric clinical trials been completed, leading to regulatory approval of fingolimod by both the United States Food and Drug Administration and the European Medicines Agency, and dimethyl fumarate and teriflunomide by the European Medicines Agency. Still, however, no highly effective monoclonal antibody therapies are approved for children with MS.^1,7^

Treatment persistence can act as an indirect global indicator of how a drug performs in routine clinical practice, reflecting a combination of effectiveness, tolerability and safety.^8,9^ Furthermore, maintaining a consistent DMT reduces the risk of rebound disease activity and offers emotional benefits by eliminating worries about efficacy, routine changes and switching risks.^10–12^ Compared to adult-onset MS,^13,14^ population-based studies comparing treatment outcomes, including treatment persistence, across different DMTs in PoMS are rare.^1^ Hence, the objective of this study was to measure treatment persistence for different DMTs in PoMS across Sweden, a country with a relatively high occurrence of PoMS.^15^ Sweden also provides a unique opportunity to compare also with B cell-depleting therapies (BCDT), since rituximab serves as an effective off-label alternative,^13,14,16^ and has become the most frequent DMT option in the adult Swedish MS population.^17^ This is also reflected by increasing use in the PoMS population during the last decade.^18^

## Methods

### Data source

We used data from the Swedish MS registry, which prospectively collects clinical data from all 64 neurology clinics across Sweden. It is estimated to capture 85% of all MS cases nationwide^19^ and has been validated specifically for the PoMS population.^20^

### Study population and inclusion criteria

Individuals were included in the study if they met all of the following criteria:

1. Clinical onset of MS before 18 years of age;
2. An initial relapsing-remitting disease course;
3. Treated with a DMT;
4. First treatment initiation before the age of 20;
5. First treatment initiation between 1 January 2000 and 30 April 2024;
6. At least one follow-up visit after the first treatment initiation.

### Standard protocol approvals, registrations and patient consents

The study was approved by the Stockholm Regional Ethical Review Board (Dnr 2017/1378-31). All individual data from the MS Registry was anonymised.

### Variables

Demographic and clinical characteristics analysed for association with subsequent treatment discontinuation included age and MS duration at treatment start, sex, Expanded Disability Status Scale (EDSS) score (last recorded within a year before each treatment initiation), healthcare region and calendar year of treatment start. Age was analysed as a continuous variable and dichotomised as a binary variable (before and after turning 20 years), sex and healthcare region as categorical variables, while EDSS was analysed as an ordinal variable. Calendar year was analysed as a continuous variable but also categorised into three epochs: the era of platform injectables from 2000 to 2006, orals and infusions from 2007 to 2018 and the pandemic/post-pandemic epoch from 2019 to 2024. The reasons for stopping therapy were analysed as a categorical variable. Finally, transitions between healthcare regions during treatment episodes were analysed as a binary variable.

### Measurements

Treatment persistence was analysed using survival analysis, modelling the time from treatment initiation to either treatment discontinuation or censoring at the last known follow-up. Treatment episodes where the last known follow-up occurred on the same day as treatment initiation were excluded. If a second or subsequent therapy was initiated during follow-up, it was included in the analysis as a new therapy start. In instances of overlapping treatment episodes, the stop date of the previous episode was adjusted to match the start date of the next episode. In alignment with similar previous studies, treatment interruptions lasting less than a month, followed by recommencement with the same therapy, were considered uninterrupted.^8^ All interferon products and glatiramer acetate were grouped as injectables. Only therapies with at least 20 treatment episodes were included in the analyses, which included injectables, dimethyl fumarate, fingolimod, natalizumab and rituximab.

### Statistical analyses

Cohort characteristics and reasons for discontinuing therapy were summarised descriptively. Continuous variables were reported as mean (standard deviation [SD]) or median (range or interquartile range [IQR]), as appropriate.

We employed a Kaplan–Meier model to estimate median treatment persistence times with 95% confidence intervals (CIs). We used Cox proportional hazards models to compare the hazard rates of treatment discontinuation across DMTs. To enhance interpretation, hazard ratios (HRs) are presented with both injectables (traditional first-line therapies) and rituximab (a highly effective DMT, the most used in this cohort) as reference groups. These estimates were obtained by re-fitting the same Cox model, re-levelling the treatment variable to alternate the reference group while keeping the covariate adjustments and model specifications identical.

The potential confounding variables of age, sex, calendar year, treatment epoch, healthcare region, EDSS score and MS duration were examined using sequential regression models. The proportional hazards assumption was assessed using Schoenfeld residuals.

Missing data were handled by complete case analysis, and no imputation was performed.

Statistical analyses and data processing were performed in R version (V.4.4.2, R Foundation for Statistical Computing, Vienna, Austria). Significance was set at a *p*-value of < 0.05.

### Sensitivity analyses

Seven pre-specified sensitivity analyses were performed. Analyses were restricted by treatment sequence (first, second, or third‑or‑later episode) and to individuals initiating their first DMT before age 18. EDSS was included as a covariate where available. Regional effects were addressed by adjusting for healthcare region in episodes without transitions and by including a binary indicator of region transition during follow‑up.

## Results

### Study population

Of 996 individuals with MS onset before age 18, 19 were excluded due to a non-relapsing–remitting course. Among the 977 remaining, 166 had not received a DMT. Of the 811 treated individuals, 403 initiated their first DMT at age 20 or later. Of the 408 who met the age and treatment criteria, 16 were excluded because they initiated treatment outside the study period, and nine were excluded due to a lack of follow-up data. Applying all inclusion criteria, the final cohort comprised 383 individuals. Cohort characteristics are summarised in Table 1.

**Table 1.** Cohort characteristics: individuals with paediatric-onset multiple sclerosis in Sweden from 2000 to 2024.

	Overall (*N* = 383)
**Age at onset, years**	
Median (IQR)	15.8 (14.3, 17.0)
**Age at first DMT start, years**	
Median (IQR)	17.2 (15.8, 18.0)
**Sex**, *N* **(%)**	
Female	265 (69.2%)
Male	118 (30.8%)
**Follow-up period, years**	
Median (IQR)	10.1 (5.5, 15.5)
**Calendar year at first DMT start**, *N* **(%)**	
2000–2006	89 (23.2%)
2007–2018	234 (61.1%)
2019–2024	60 (15.7%)
**Total number of DMTs**, *N* **(%)**	
One	127 (33.2%)
Two	95 (24.8%)
Three	85 (22.2%)
Four or more	76 (19.8%)

IQR: interquartile range, DMT: disease-modifying therapy.

### Treatment episodes

**Figure 1.**
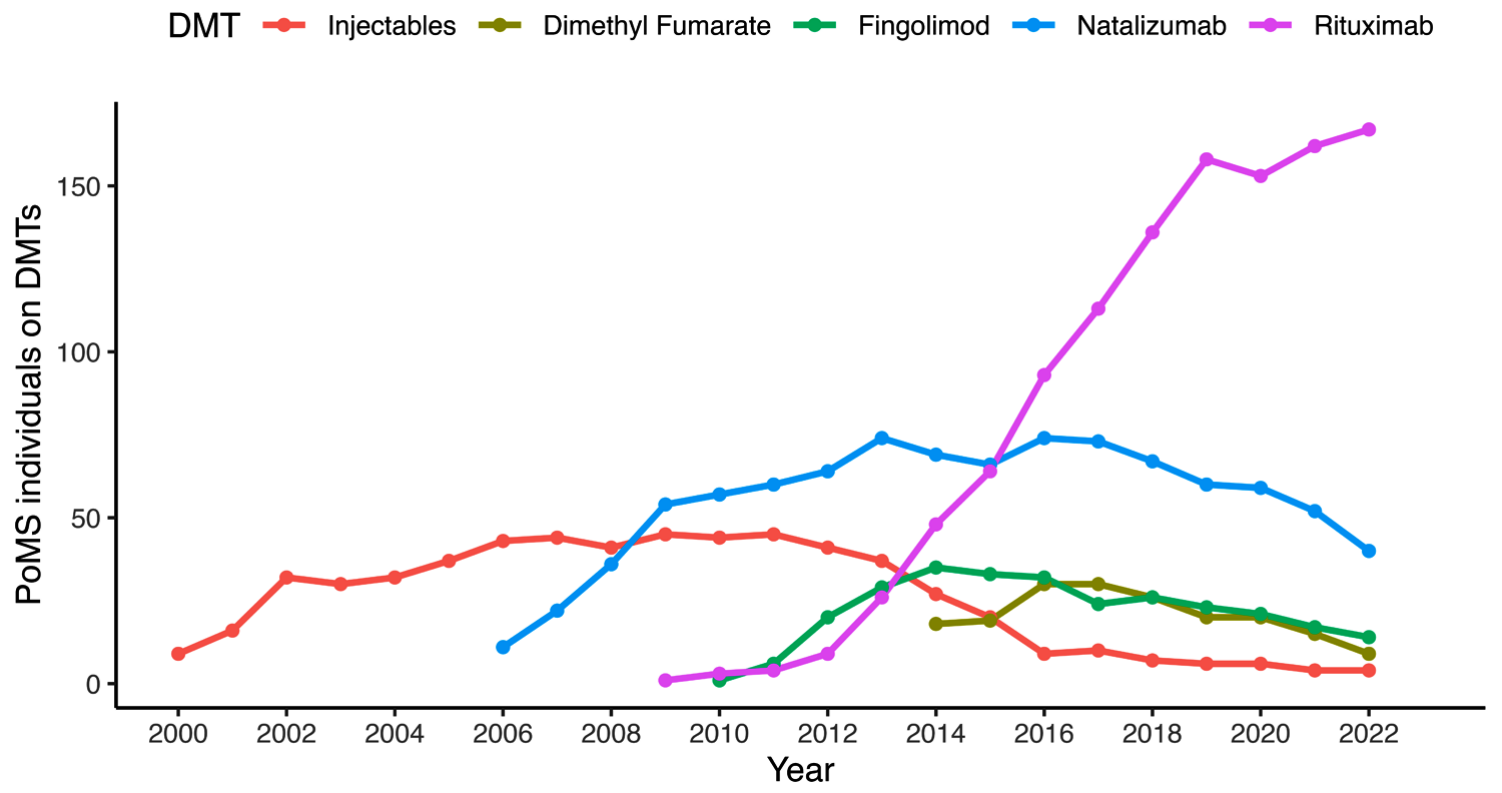
Annual number of individuals with paediatric-onset multiple sclerosis in Sweden, categorised by ongoing disease-modifying therapy from 2000 to 2022.

**Figure 2.**
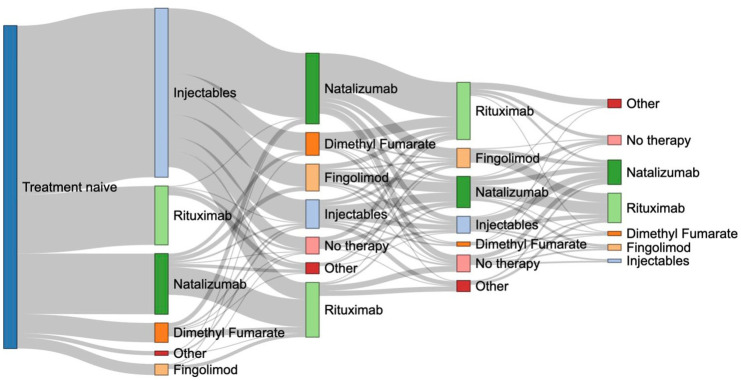
Sankey diagram illustrating the pattern of switches between therapies for 383 individuals with paediatric-onset multiple sclerosis who received disease-modifying therapies from 2000 to 2024. The first four treatment episodes for each individual in the cohort and the transitions between these episodes are depicted. A grey line represents a switch from one therapy to another.

Throughout the entire study period, 934 treatment episodes were recorded. Overall, rituximab was the most used DMT (*N* = 272), followed by injectables (*N* = 263), natalizumab (*N* = 248), fingolimod (*N* = 86) and dimethyl fumarate (*N* = 65) (Table 2). Injectables were the only prescribed DMTs until the introduction of natalizumab in 2006, followed by rituximab in 2009, fingolimod in 2010 and dimethyl fumarate in 2014 (Figure 1). The median age at initiation of any DMT, regardless of treatment sequence, was 18.9 years, and when stratified by DMT, ranged from 17.7 years for injectables to 22.2 years for fingolimod. The sex distribution varied, showing a female predominance ranging from 65.1% for fingolimod to 75.3% for injectables. The median MS duration at treatment start ranged from 1.3 years for injectables to 6.6 years for fingolimod. The median EDSS value at treatment start ranged from 1 to 2 across all DMTs, with data available for approximately two-thirds of treatment episodes. Missing data were more common among platform injectables, which were predominantly used earlier in the study period. The distribution of DMTs varied slightly across healthcare regions, showing a similar pattern of missingness as with EDSS. Transitions between regions during follow-up occurred in 39 treatment episodes. In individuals with PoMS who were treated with injectables, these drugs were primarily prescribed as the first DMT, while the other DMTs were mostly used as a second or subsequent option (Table 2 and Figure 2). Characteristics of the initial therapy episodes for each individual are summarised in eTable 1, comprising a total of 383 episodes. These included 203 episodes for injectables, 73 for natalizumab, 71 for rituximab, 23 for dimethyl fumarate and 13 for fingolimod.

**Table 2.** Characteristics at the start of each treatment episode for individuals with paediatric-onset multiple sclerosis in Sweden from 2000 to 2024.

	Injectables	Dimethyl fumarate	Fingolimod	Natalizumab	Rituximab	Overall
	(*N* = 263)	(*N* = 65)	(*N* = 86)	(*N* = 248)	(*N* = 272)	(*N* = 934)
Age, years						
Median (IQR)	17.7 (16.2, 18.8)	19.4 (17.7, 24.4)	22.2 (18.8, 25.8)	19.4 (17.2, 24.7)	20.7 (17.6, 25.6)	18.9 (17.1, 23.7)
**Sex**, *N* **(%)**						
Female	198 (75.3%)	43 (66.2%)	56 (65.1%)	182 (73.4%)	195 (71.7%)	674 (72.2%)
Male	65 (24.7%)	22 (33.8%)	30 (34.9%)	66 (26.6%)	77 (28.3%)	260 (27.8%)
**MS duration, years**						
Median (IQR)	1.3 (0.5, 3.9)	3.7 (1.4, 8.5)	6.6 (2.8, 11.0)	4.4 (1.2, 9.4)	5.2 (1.5, 10.7)	3.5 (1.0, 8.3)
**EDSS, value**						
Median (IQR)	1.5 (0.0, 2.0)	1.0 (0.0, 1.0)	1.0 (0.0, 2.0)	2.0 (1.0, 2.5)	1.5 (0.0, 2.0)	1.5 (0.0, 2.0)
Missing	161 (61.2%)	29 (44.6%)	24 (27.9%)	60 (24.2%)	100 (36.8%)	374 (40.0%)
**Healthcare Region**, *N* **(%)**						
Middle	24 (9.1%)	9 (13.8%)	7 (8.1%)	33 (13.3%)	41 (15.1%)	114 (12.2%)
North	18 (6.8%)	< 5	9 (10.5%)	27 (10.9%)	47 (17.3%)	105 (11.2%)
South	11 (4.2%)	8 (12.3%)	11 (12.8%)	21 (8.5%)	22 (8.1%)	73 (7.8%)
Southeast	7 (2.7%)	-	7 (8.1%)	12 (4.8%)	23 (8.5%)	55 (5.9%)
Stockholm	47 (17.9%)	14 (21.5%)	18 (20.9%)	44 (17.7%)	48 (17.6%)	171 (18.3%)
West	11 (4.2%)	9 (13.8%)	13 (15.1%)	47 (19.0%)	32 (11.8%)	112 (12.0%)
Missing	145 (55.1%)	15 (23.1%)	21 (24.4%)	64 (25.8%)	59 (21.7%)	304 (32.5%)
**DMT sequence**, *N* **(%)**						
First DMT	203 (77.2%)	23 (35.4%)	13 (15.1%)	73 (29.4%)	71 (26.1%)	383 (41.0%)
Second or subsequent DMT	60 (22.8%)	42 (64.6%)	73 (84.9%)	175 (70.6%)	201 (73.9%)	551 (59.0%)
**Treatment epoch**, *N* **(%)**						
2000–2005	89 (33.8%)	0 (0%)	0 (0%)	0 (0%)	0 (0%)	89 (9.5%)
2006–2018	162 (61.6%)	58 (89.2%)	79 (91.9%)	208 (83.9%)	161 (59.2%)	668 (71.5%)
2019–2024	12 (4.6%)	7 (10.8%)	7 (8.1%)	40 (16.1%)	111 (40.8%)	177 (19.0%)

To protect confidentiality, cell sizes < 5 were masked. Additional cells were suppressed to prevent back-calculation of masked values as needed. IQR: interquartile range; DMT: disease-modifying therapy; EDSS: Expanded Disability Status Scale.

### Treatment trajectories

Most of the cohort (66.8%) switched DMT at least once during the study. When broken down by DMT, this was true for all DMTs except rituximab. This pattern appeared regardless of whether the DMT was used as the first, second or later treatment in the sequence. Figure 2 shows a Sankey diagram illustrating the first four treatment episodes for each individual and the transitions between them.

### Treatment persistence

**Figure 3.**
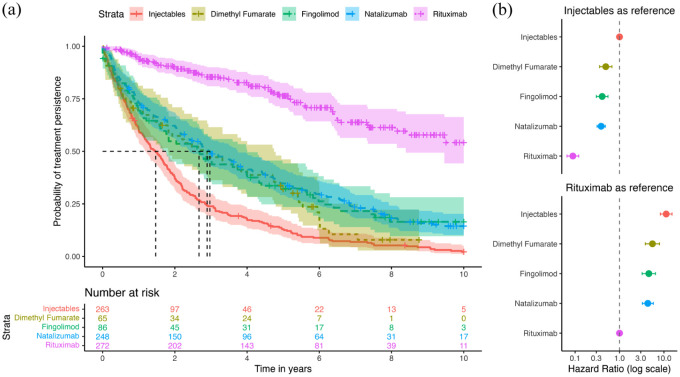
Probability of treatment persistence in paediatric-onset multiple sclerosis in Sweden from 2000 to 2024. To the left (a), treatment persistence is visualised in a Kaplan–Meier plot. The dashed lines represent estimated median treatment persistence. Notably, rituximab did not decline to the median. To the right (b), adjusted hazard ratios for discontinuing therapy, along with 95% confidence intervals, are provided, using injectables and rituximab as the reference.

In the Kaplan–Meier analysis, the median persistence across all DMTs was 37.5 months (95% CI: 33.3–44.8). When stratified by treatment, notable differences appeared. Rituximab displayed a distinct pattern, with persistence estimates not declining to the median during follow-up. Among the other DMTs, natalizumab exhibited the longest median persistence at 35.6 months (95% CI: 28.8–44.9), followed by dimethyl fumarate (34.7 months; 95% CI: 21.7–56.9), fingolimod (32.0 months; 95% CI: 21.1–48.1) and injectables (17.6 months; 95% CI: 14.4–20.8) (Figure 3).

Consistent with the absolute differences, the Cox regression model revealed significant variation in the risk of treatment discontinuation. In the unadjusted model, compared to injectables, rituximab was associated with a markedly lower risk (HR: 0.14; 95% CI: 0.11–0.18), as were natalizumab (HR: 0.51; 95% CI: 0.43–0.62), fingolimod (HR: 0.55; 95% CI: 0.42–0.72) and dimethyl fumarate (HR: 0.64; 95% CI: 0.47–0.86). After adjusting for age (continuous), sex and treatment epoch, the associations remained robust, with somewhat larger effect sizes. Adjusted hazard ratios ranged from 0.09 (95% CI: 0.07–0.12) for rituximab to 0.49 (95% CI: 0.36–0.68) for dimethyl fumarate.

When using rituximab as the reference group, all other DMTs were associated with a significantly increased risk of discontinuation in both unadjusted and adjusted models. Unadjusted HRs ranged from 3.70 (95% CI: 2.82–4.87) for natalizumab to 7.22 (95% CI: 5.53–9.44) for injectables, while adjusted HRs ranged from 4.33 (95% CI: 3.26–5.76) for natalizumab to 11.23 (95% CI: 8.30–15.20) for injectables (eTable 2, eTable 3, and Figure 3).

#### Sensitivity analyses

All pre-specified sensitivity analyses supported the main findings. In the primary sensitivity analysis restricted to each individual’s first treatment episode, rituximab was associated with an even more pronounced reduction in discontinuation risk compared with other DMTs (eTable 2 and eTable 3).

### Reasons for stopping therapy

During the study period, 543 of the 934 treatment episodes (58%) were discontinued. Reasons for discontinuation differed by therapy. Insufficient treatment effect was the most common reason for discontinuation of injectables (40.3%), dimethyl fumarate (50.0%) and fingolimod (45.5%). JC virus positivity accounted for 38.7% of natalizumab discontinuations, while side effects were the leading reason for rituximab (34.8%). Further details are presented in Table 3.

**Table 3.** Reasons for stopping therapy among individuals with paediatric-onset multiple sclerosis in Sweden from 2000 to 2024.

	Injectables (*N* = 258)	Dimethyl Fumarate (*N* = 52)	Fingolimod (*N* = 66)	Natalizumab (*N* = 204)	Rituximab (*N* = 69)
**Reason for discontinuing therapy**, *N* **(%)**					
Insufficient effect	104 (40.3%)	26 (50.0%)	30 (45.5%)	-	9 (13.0%)
Side effects	78 (30.2%)	13 (25.0%)	15 (22.7%)	12 (5.9%)	24 (34.8%)
Pregnancy/planned pregnancy	15 (5.8%)	6 (11.5%)	9 (13.6%)	38 (18.6%)	11 (15.9%)
JC virus	0 (0%)	0 (0%)	0 (0%)	79 (38.7%)	< 5
Other	51 (19.8%)	7 (13.5%)	12 (18.2%)	69 (33.8%)	23 (33.3%)
Missing	10 (3.9%)	0 (0%)	0 (0%)	< 5	-

For natalizumab, 6 of 69 discontinuations categorised as ‘Other’ reflected planned bridging to allow for vaccination before initiating rituximab. To protect confidentiality, cell sizes < 5 were masked. Additional cells were suppressed to prevent back-calculation of masked values as needed.

## Discussion

In this population-based cohort study of 383 individuals with PoMS, treatment persistence with rituximab was significantly higher relative to all other DMTs. This pattern was evident in the primary analysis, which included all therapy episodes per individual, and was even more pronounced when only the first therapy episode for each individual was measured. As expected, the difference was most marked in comparison to injectables. However, substantial and consistent differences were also observed when compared to dimethyl fumarate and fingolimod, both approved for paediatric use, as well as natalizumab, a highly effective monoclonal antibody therapy used off-label in PoMS.^7,21^ The reasons for stopping therapy varied among the therapy groups. Insufficient treatment effect was the most common reason for discontinuation of injectables, dimethyl fumarate and fingolimod. For natalizumab, the leading cause was JC virus positivity, indicating increased risk of progressive multifocal leukoencephalopathy, while side effects were the most common reason for discontinuing rituximab.

Comparative treatment persistence in PoMS has previously been studied. In a large observational study using the MSBase registry, comparing injectables, fingolimod and natalizumab, the time to treatment discontinuation was significantly longer for patients treated with fingolimod (HR: 0.24; 95% CI: 0.15–0.39) or natalizumab (HR: 0.24, 95% CI: 0.16–0.36) than for those treated with injectables. In contrast, there was no difference between patients treated with fingolimod and those treated with natalizumab.^4^ In a French multicentre observational study, the HR of discontinuing moderately effective therapies (including, among other DMTs, injectables) was 5.97 (95% CI: 2.92–12.20) after 2 years of treatment compared with highly effective therapies (including, among other DMTs, fingolimod, natalizumab and rituximab). However, in this study, no within-group analyses were performed.^22^ In a UK-wide observational study comparing injectables to newer oral and infusion therapies (including dimethyl fumarate, fingolimod, natalizumab among others), the treatment persistence was higher for newer therapies.^3^ In an Italian multicentre study comparing fingolimod to natalizumab, there was no difference in treatment persistence between the two groups.^23^ All these studies are thus in line with our results.

BCDT has proven to be both effective and relatively safe^24^ for both adults^13,14,16^ and children^18,25^ with MS. In Sweden, rituximab has become increasingly popular during the last decade and is now prescribed to the majority of all individuals with MS.^17^ This was initiated shortly after publication of a first promising phase II trial in relapsing-remitting MS in 2008.^26^ However, BCDT formally approved for MS came only in 2017 with ocrelizumab, and now also includes ofatumumab (2020) and ublituximab (2022/2023).^27^ Importantly, the World Health Organization (WHO) has included rituximab on their essential medicines list, as it represents a cost-effective BCDT treatment option that is available worldwide.^28^ Still, there is no BCDT specifically approved for PoMS, but there are currently two ongoing trials, one of ocrelizumab^29^ and one of ofatumumab,^30^ both using fingolimod as the comparator. Comparative evidence on treatment persistence for BCDTs in PoMS remains limited. To the best of our knowledge, beyond the French multicentre study that included seven individuals treated with rituximab, four with ocrelizumab and one with ofatumumab, all grouped under highly effective therapies,^22^ no further comparative studies have been published. One large American PoMS cohort study was descriptive but offers some comparative context. In that study, nearly half of 166 rituximab-treated individuals discontinued therapy over a median follow-up of 1.5 years, contrasting with our findings. Discontinuation proportions were similar for natalizumab and dimethyl fumarate. Proportions were lower for fingolimod and markedly lower for ocrelizumab (≈10%), which was comparable to rituximab in our data. Most rituximab discontinuations were classified under ‘other’ reasons, and only a few were due to inefficacy or adverse events.^25^ It is not possible to determine from the data what these ‘other’ reasons were, but it may reflect access-related challenges or transitions to ocrelizumab. Our findings clearly indicate that the real-world treatment persistence for rituximab significantly exceeds that of all other DMTs. This suggests a more favourable risk-benefit profile compared to other DMTs. In addition, maintaining a consistent DMT reduces the risk of rebound disease activity associated with discontinuing and switching from DMTs like natalizumab^11^ and fingolimod.^12^ Furthermore, continuing therapy offers emotional benefits by eliminating concerns about efficacy, changes in administration routines and the risk profile associated with switching.^10^ In summary, our findings align with similar studies in AoMS,^13,14^ corroborate previous data in PoMS,^18,25^ supporting early consideration of BCDTs in PoMS.

### Strengths and limitations

Sweden is a prime setting for epidemiological research, with nationwide, population-based registers, like the MS registry covering 85% of MS cases.^19^ The registry has recently been validated for the PoMS population.^20^ In addition, Sweden has maintained a tradition of treating individuals with MS, both children and adults, using rituximab for over a decade.^17,18^ This provides a somewhat unique opportunity to analyse a large PoMS cohort with comparative long-term treatment data, including rituximab use.

The observational design of the study is inherently susceptible to confounding. On the other hand, observational studies can yield significant insights regarding the comparative performance of drugs in a real-world context, which is often absent in RCTs.^31,32^ We conducted sensitivity analyses addressing treatment sequencing and disability. Results from these analyses aligned with the main analysis, providing support that confounding by indication was not a major concern. Another possible consideration relates to the age at first treatment initiation. Previous studies on treatment persistence in PoMS restricted inclusion to individuals initiating treatment before age 18,^3,4,22,23^ whereas we allowed initiation up to age 20 to account for diagnostic delay. However, the sensitivity analysis restricted to first DMT initiation before age 18 yielded highly consistent results. Moreover, natalizumab is occasionally used as a short-term bridging therapy to enable vaccination before initiating rituximab.^33,34^ This practice may contribute to early discontinuation of natalizumab and could potentially affect treatment persistence estimates. This reason was given for 3% of natalizumab discontinuations. While this may partly explain the initial decline observed in natalizumab persistence (Figure 3), the continued reduction in persistence throughout follow-up suggests that this effect is unlikely to have substantially affected the overall findings. Further, the high persistence for rituximab may, in some cases, be inflated by rituximab being the last-line therapy rather than the superior alternative. However, our sensitivity analysis that restricted to only the first therapy per individual showed an even more pronounced improvement in persistence with rituximab than the main model including subsequent episodes. While treatment persistence serves as a useful proxy for real-world effectiveness and safety, this study did not assess direct clinical outcomes such as relapse rates, MRI activity or adverse events. These areas require further study. Also, safety monitoring of BCDTs requires extended follow-up. Reassuringly, the follow-up time for rituximab treatment in our cohort was relatively long, with a median of 4.1 years and individual follow-up extending to nearly 15 years. Nonetheless, long‑term immunological consequences such as hypogammaglobulinaemia and infection require further characterisation. Furthermore, specific reasons for discontinuing DMTs, especially natalizumab and rituximab, were missing in up to a third of all episodes, limiting the scope of further analysis. Moreover, discontinuation may occur for reasons unrelated to effectiveness or safety, which were not fully captured in this analysis. Nevertheless, the extent and consistency of the differences in persistence, especially favouring rituximab, indicate a favourable benefit–risk profile.

## Conclusion

Treatment persistence was significantly higher with newer oral and infusion DMTs compared to injectables. Rituximab demonstrated markedly greater persistence than all other DMTs, supporting BCDT use in PoMS. Further long-term studies are needed to clarify the comparative effectiveness and safety profiles of these treatments.

## Supplemental Material

sj-docx-1-msj-10.1177_13524585261448789 – Supplemental material for Treatment persistence in paediatric-onset multiple sclerosis: A Swedish nationwide registry study Supplemental material, sj-docx-1-msj-10.1177_13524585261448789 for Treatment persistence in paediatric-onset multiple sclerosis: A Swedish nationwide registry study by Fredrik Sandesjö, Kyla A McKay, Katharina Fink, Fredrik Piehl and Ronny Wickström in Multiple Sclerosis Journal
