# Supplementary material for: Treatment persistence in paediatric-onset multiple sclerosis: A Swedish nationwide registry study
Source: Mult Scler. 2026 May 31;32(8):829–38. doi: 10.1177/13524585261448789 (PMC13333028; doi:10.1177/13524585261448789)
Supplement: sj-docx-1-msj-10.1177_13524585261448789 – Supplemental material for Treatment persistence in paediatric-onset multiple sclerosis: A Swedish nationwide registry study [file sj-docx-1-msj-10.1177_13524585261448789.docx]

|  | Injectables (N=203) | Dimethyl Fumarate (N=23) | Fingolimod (N=13) | Natalizumab (N=73) | Rituximab (N=71) | Overall (N=383) |
| --- | --- | --- | --- | --- | --- | --- |
| **Age, years** |  |  |  |  |  |  |
| Median (IQR) | 17.2 (16.0, 18.0) | 17.9 (16.9, 18.8) | 17.8 (16.0, 18.4) | 17.0 (15.6, 17.8) | 17.1 (15.6, 17.9) | 17.2 (15.8, 18.0) |
| **Sex, N (%)** |  |  |  |  |  |  |
| Female | 144 (70.9%) | 15 (65.2%) | - | 47 (64.4%) | 49 (69.0%) | 265 (69.2%) |
| Male | 59 (29.1%) | 8 (34.8%) | <5 | 26 (35.6%) | 22 (31.0%) | 118 (30.8%) |
| **MS duration, years** |  |  |  |  |  |  |
| Median (IQR) | 0.9 (0.4, 1.9) | 1.6 (0.5, 2.6) | 1.6 (1.1, 2.0) | 0.6 (0.2, 1.9) | 0.4 (0.2, 1.7) | 0.7 (0.3, 2.0) |
| **EDSS, value** |  |  |  |  |  |  |
| Median (IQR) | 1.3 (0.0, 2.0) | 1.0 (0.0, 1.0) | 0.0 (0.0, 0.0) | 1.5 (0.0, 2.0) | 1.5 (0.0, 2.0) | 1.5 (0.0, 2.0) |
| Missing | 135 (66.5%) | 12 (52.2%) | 8 (61.5%) | 24 (32.9%) | 38 (53.5%) | 217 (56.7%) |
| **Calendar year, N (%)** |  |  |  |  |  |  |
| 2000–2005 | - | 0 (0%) | 0 (0%) | 0 (0%) | 0 (0%) | 74 (19.3%) |
| 2006–2018 | 125 (61.6%) | - | - | 56 (76.7%) | 35 (49.3%) | 249 (65.0%) |
| 2019–2024 | <5 | <5 | <5 | 17 (23.3%) | 36 (50.7%) | 60 (15.7%) |
| eTable 1. Characteristics at the start of the first treatment episode for individuals with pediatric-onset multiple sclerosis in Sweden from 2000 to 2024. To protect confidentiality, cell sizes <5 were masked. Additional cells were suppressed to prevent back-calculation of masked values as needed.IQR = interquartile range, DMT = Disease-modifying therapy, EDSS = Expanded Disability Status Scale. | | | | | | |

| Model | Therapy Reference | Dimethyl Fumarate | Fingolimod | Natalizumab | Rituximab |
| --- | --- | --- | --- | --- | --- |
| Unadjusted | Injectables | 0.64 (0.47–0.86) | 0.55 (0.42–0.72) | 0.51 (0.43–0.62) | 0.14 (0.11–0.18) |
| Age | Injectables | 0.59 (0.43–0.80) | 0.48 (0.36–0.64) | 0.48 (0.40–0.58) | 0.12 (0.09–0.16) |
| Age + Sex | Injectables | 0.60 (0.44–0.82) | 0.49 (0.37–0.66) | 0.48 (0.40–0.59) | 0.12 (0.09–0.17) |
| Age + Sex + Treatment epoch | Injectables | 0.49 (0.36–0.68) | 0.41 (0.30–0.55) | 0.39 (0.31–0.48) | 0.09 (0.07–0.12) |
|  |  |  |  |  |  |
| Sensitivity analyses |  |  |  |  |  |
| First therapy only (n = 383) Age + Sex + Treatment epoch | Injectables | 0.43 (0.25–0.72) | 0.26 (0.12–0.56) | 0.30 (0.21–0.42) | 0.04 (0.02–0.09) |
| Second therapy only (n = 256) Age + Sex + Treatment epoch | Injectables | 0.47 (0.26–0.85) | 0.37 (0.20–0.67) | 0.26 (0.16–0.42) | 0.09 (0.04–0.16) |
| Third or later therapy only (n = 295) Age + Sex + Treatment epoch | Injectables | 0.54 (0.26–1.13) | 0.44 (0.25–0.78) | 0.55 (0.33–0.91) | 0.11 (0.06–0.19) |
| First therapy < 18 years (n = 666) Age + Sex + Treatment epoch | Injectables | 0.60 (0.40–0.89) | 0.47 (0.33–0.68) | 0.39 (0.30–0.50) | 0.10 (0.07–0.14) |
| Adjusting for EDSS (n = 560) Age + Sex + Treatment epoch + EDSS | Injectables | 0.39 (0.25–0.62) | 0.42 (0.28–0.61) | 0.38 (0.28–0.51) | 0.10 (0.07–0.14) |
| Adjusting for region (no transitions, n = 591) Age + Sex + Treatment epoch + Healthcare Region | Injectables | 0.58 (0.39–0.87) | 0.50 (0.34–0.73) | 0.43 (0.33–0.57) | 0.11 (0.07–0.15) |
| Adjusting for Healthcare Region transitions (n = 630) Age + Sex + Treatment epoch + Healthcare Region Transition | Injectables | 0.51 (0.35–0.77) | 0.49 (0.34–0.71) | 0.43 (0.33–0.57) | 0.11 (0.08–0.16) |
| eTable 2. Hazard ratios for stopping therapy using Injectables as reference. Unadjusted and cumulatively adjusted estimates are presented with 95% confidence intervals. Sensitivity analyses are based on the fully adjusted model including age, sex, treatment epoch, and additional covariates as specified. For the sensitivity analyses, n denotes the number of treatment episodes included in each analytic subset. EDSS: Expanded Disability Status Scale | | | | | |

| Model | Therapy Reference | Injectables | Dimethyl Fumarate | Fingolimod | Natalizumab |
| --- | --- | --- | --- | --- | --- |
| Unadjusted | Rituximab | 7.22 (5.53–9.44) | 4.62 (3.22–6.63) | 3.98 (2.84–5.57) | 3.70 (2.82–4.87) |
| Age | Rituximab | 8.13 (6.13–10.79) | 4.77 (3.32–6.84) | 3.91 (2.79–5.49) | 3.92 (2.97–5.17) |
| Age + Sex | Rituximab | 8.04 (6.06–10.67) | 4.83 (3.36–6.94) | 3.97 (2.83–5.56) | 3.88 (2.94–5.12) |
| Age + Sex + Treatment epoch | Rituximab | 11.23 (8.30–15.20) | 5.51 (3.81–7.96) | 4.57 (3.23–6.46) | 4.33 (3.26–5.76) |
|  |  |  |  |  |  |
| Sensitivity analyses |  |  |  |  |  |
| First therapy only (n = 383) Age + Sex + Treatment epoch | Rituximab | 22.93 (11.41–46.07) | 9.77 (4.30–22.18) | 5.93 (2.22–15.88) | 6.81 (3.39–13.67) |
| Second therapy only (n = 256) Age + Sex + Treatment epoch | Rituximab | 11.64 (6.07–22.34) | 5.48 (2.87–10.48) | 4.32 (2.29–8.13) | 2.98 (1.72–5.14) |
| Third or later therapy only (n = 295) Age + Sex + Treatment epoch | Rituximab | 9.09 (5.26–15.73) | 4.95 (2.55–9.62) | 3.99 (2.49–6.38) | 4.99 (3.35–7.43) |
| First therapy < 18 years (n = 666) Age + Sex + Treatment epoch | Rituximab | 10.13 (7.12–14.40) | 6.05 (3.87–9.45) | 4.80 (3.17–7.25) | 3.91 (2.81–5.45) |
| Adjusting for EDSS (n = 560) Age + Sex + Treatment epoch + EDSS | Rituximab | 10.13 (6.90–14.88) | 3.99 (2.45–6.48) | 4.21 (2.82–6.29) | 3.86 (2.78–5.38) |
| Adjusting for region (no transitions, n = 591) Age + Sex + Treatment epoch + Healthcare Region | Rituximab | 9.50 (6.59–13.70) | 5.54 (3.61–8.50) | 4.76 (3.17–7.12) | 4.12 (2.97–5.72) |
| Adjusting for Healthcare Region transitions (n = 630) Age + Sex + Treatment epoch + Healthcare Region Transition | Rituximab | 9.07 (6.35–12.95) | 4.67 (3.08–7.07) | 4.43 (3.03–6.49) | 3.91 (2.86–5.34) |
| eTable 3. Hazard ratios for stopping therapy using Rituximab as reference. Unadjusted and cumulatively adjusted estimates are presented with 95% confidence intervals. Sensitivity analyses are based on the fully adjusted model including age, sex, treatment epoch, and additional covariates as specified. For the sensitivity analyses, n denotes the number of treatment episodes included in each analytic subset. EDSS: Expanded Disability Status Scale | | | | | |
